# An integrated centrifugation-washing-lysis method significantly improves microbiological diagnosis of spontaneous bacterial peritonitis

**DOI:** 10.1128/spectrum.03435-25

**Published:** 2026-03-16

**Authors:** Yihe Zhao, Mingxi Hua, Chen Chen, Qinghui Zhuang, Yu Hao, Yu Cao, Hengkun Wei, Chenchen Wang, Jingyuan Liu, Hui Zeng, Ang Li

**Affiliations:** 1 Intensive Care Unit, Beijing Ditan Hospital, Capital Medical University 12517, Beijing, China; 2 Biomedical Innovation Center, Beijing Shijitan Hospital, Capital Medical University 12517, Beijing, China; 3 Beijing Key Laboratory for Therapeutic Cancer Vaccines, Beijing Shijitan Hospital, Capital Medical University 12517, Beijing, China; Michigan State University, East Lansing, Michigan, USA

**Keywords:** diagnostics, spontaneous bacterial peritonitis, leukocyte lysis, bacterial culture

## Abstract

Culture-negative spontaneous bacterial peritonitis (SBP) remains a major diagnostic challenge in patients with cirrhotic ascites, largely due to prior antibiotic exposure, low bacterial burden, and phagocytosis of bacteria. To address these factors and reduce false-negative results, we developed an integrated centrifugation-washing-lysis method and evaluated its impact on diagnostic turnaround time and antimicrobial stewardship. Ascitic fluid was concentrated and washed, followed by selective leukocyte lysis with 0.5% Triton X-100 (optimized to lyse host cells while preserving bacterial viability during 1-min incubation). The resulting pellet was inoculated onto solid media (chocolate and blood agar). This method was prospectively evaluated against standard bedside inoculation into aerobic and anaerobic blood culture bottles using 160 ascitic fluid samples from patients with clinically suspected SBP. The modified culture method demonstrated a significantly higher positivity rate (30.6% [49/160] vs 13.8% [22/160]; *P* < 0.001) and a 2.82-fold increase in total bacterial isolates (62 vs 22), revealing previously undetected monomicrobial and polymicrobial infections. Time to detection was reduced by 56.5% (1.0 day vs 2.3 days; *P* < 0.001) , with visible colony growth on solid media after a mean time of 24 h, enabling same-day organism identification and antimicrobial susceptibility testing. By releasing viable phagocytosed bacteria, the integrated centrifugation-washing-lysis method significantly improves the microbiological diagnosis of SBP by increasing culture yield, shortening time to diagnosis, and enhancing detection of polymicrobial and multidrug-resistant infections. This modified cultrue method may support earlier targeted antimicrobial therapy and improved stewardship in patients with cirrhosis.

**IMPORTANCE:**

Our research addresses the critical limitation of low positivity rates in ascitic fluid cultures for spontaneous bacterial peritonitis (SBP) diagnosis. By developing a novel ascitic fluid culture technique, we increases SBP culture positivity from 13.8% to 30.6%. This rapid and cost-effective approach not only cuts diagnosis time from 2.3 to 1 day but also reveals previously missed polymicrobial infections, enabling timely targeted therapy and reducing unnecessary antibiotic use in high-risk patients.

## INTRODUCTION

Spontaneous bacterial peritonitis (SBP) constitutes a severe and life-threatening complication in cirrhotic patients with ascites, characterized by ascitic fluid infection in the absence of an identifiable abdominal infectious source. Its prevalence ranges from 40% to 70% in this population (1, 2). SBP significantly contributes to short-term mortality and plays a key role in the pathogenesis of acute-on-chronic liver failure and acute kidney injury (3, 4). Critically, untreated SBP carries an in-hospital mortality rate as high as 50%–60% (2, 5), underscoring the imperative necessity for prompt diagnosis and intervention. While empirical antibiotic therapy remains a cornerstone of management, its widespread use presents significant challenges. Antibiotics impose metabolic burdens on compromised hepatic and renal function and contribute to the proliferation of antimicrobial resistance (6, 7). Consequently, rapid and accurate microbiological diagnosis is paramount for guiding targeted therapy and optimizing patient outcomes.

European Association for the Study of the Liver, American Association for the Study of Liver Diseases, and Chinese Society of Hepatology Practice Guidelines recommend that for patients with new-onset ascites or suspected intra-abdominal infection, at least 10 mL of ascitic fluid should be drawn at the bedside before antibiotic administration and directly inoculated into blood culture bottles (both aerobic and anaerobic) for incubation (2, 8–10). However, in clinical practice, this approach exhibits significant limitations of low sensitivity and low positive rates (10%–20%) (11), mainly due to (i) empirical antibiotic administration prior to specimen collection inhibits the bacterial growth (12); (ii) low bacterial load in ascitic fluid (13); and (iii) clearance by immune cells which was confirmed by visualization of bacterial phagocytosis by neutrophils in respiratory tract infection specimens (14–16). Moreover, the prolonged duration required for bacterial culture, coupled with delayed antimicrobial initiation, can significantly increase case fatality rates.

Recently, bacterial DNA (bactDNA) detection and sequencing have enhanced pathogen detection sensitivity with a rapid turnaround time (4–5 h) (17, 18). However, qualitative bactDNA analysis alone cannot reliably diagnose SBP, as immune-mediated bacterial translocation may release DNA fragments without active infection (19–21). Therefore, bacterial culture method remains essential for identifying viable SBP-causing pathogens.

To address the persistent diagnostic challenges posed by prior antibiotic exposure, low bacterial load, and phagocytic sequestration inherent in conventional ascitic fluid cultures, we developed a novel bacterial culture strategy. This approach integrated sample concentration and washing to enrich bacteria and remove antibiotic interference, coupled with Triton X-100-mediated selective immune cell lysis—exploiting its targeted membrane-disruptive properties to liberate viable intracellular bacteria while preserving cultivability (22). The optimized protocol significantly increased culture positivity and reduced time-to-detection compared to standard methods.

## RESULTS

### Establishment and optimization of the modified culture method

**Fig 1:**
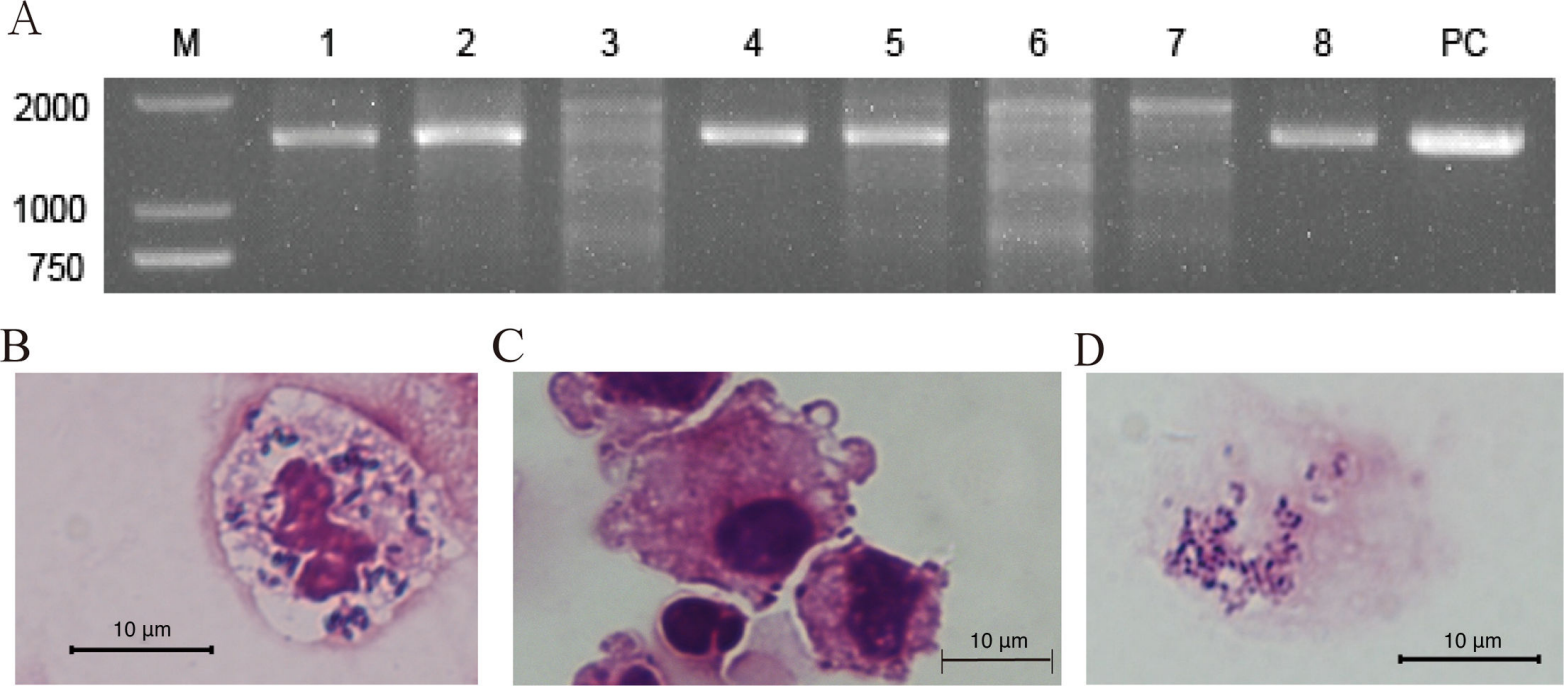
Molecular and microscopic detection of bacteria in culture-negative ascitic fluid from SBP patients. (**A**) 16S rRNA PCR amplification from ascitic fluid of eight antibiotic-responsive SBP patients with negative conventional cultures. Distinct amplification bands (lanes 1, 2, 4, 5, and 8) confirm bacterial presence in 5/8 samples. (**B–D**) Representative Gram staining showing bacterial aggregates within polymorphonuclear leukocytes (PMNs; **B**), macrophages (**C**), and Triton X-100-treated ascitic fluid samples (**D**), corroborating intracellular bacterial localization. PCR, polymerase chain reaction; rRNA, ribosomal RNA; PC, positive control; M, DNA molecular weight marker (bp).

To establish an improved bacterial culture method, we first collected eight ascitic fluid samples from eight patients with clinically suspected SBP. These patients showed a clinical response to antibiotics despite negative results from conventional cultures. To identify whether pathogenic bacteria exist, the bacterial 16S rRNA gene was amplified from extracted DNA using universal bacterial primers (27F/1492R) (23). Distinct amplification bands of full-length 16S rRNA amplicons were observed in five of the eight samples, indicating the presence of bacteria that were not detected by the conventional culture method (Fig. 1A). In addition, we subsequently performed Gram staining of the ascitic fluid and found that bacterial aggregates localized within polymorphonuclear leukocytes (PMNs) and macrophages, confirming the presence of bacteria in the culture-negative ascitic fluid samples (Fig. 1B and C). These results indicated that, in some samples that were negative by conventional culture, bacteria were present within PMNs and macrophages. The widespread phagocytosis by resident immune cells might have markedly reduced the number of detectable bacteria, thereby explaining the low culture positivity rate. To improve bacterial detection, we proposed that concentrating ascitic fluid specimens followed by treatment with Triton X-100, a cell membrane-disruptive agent, could release intracellular bacteria and enhance culture yield.

**Fig 2:**
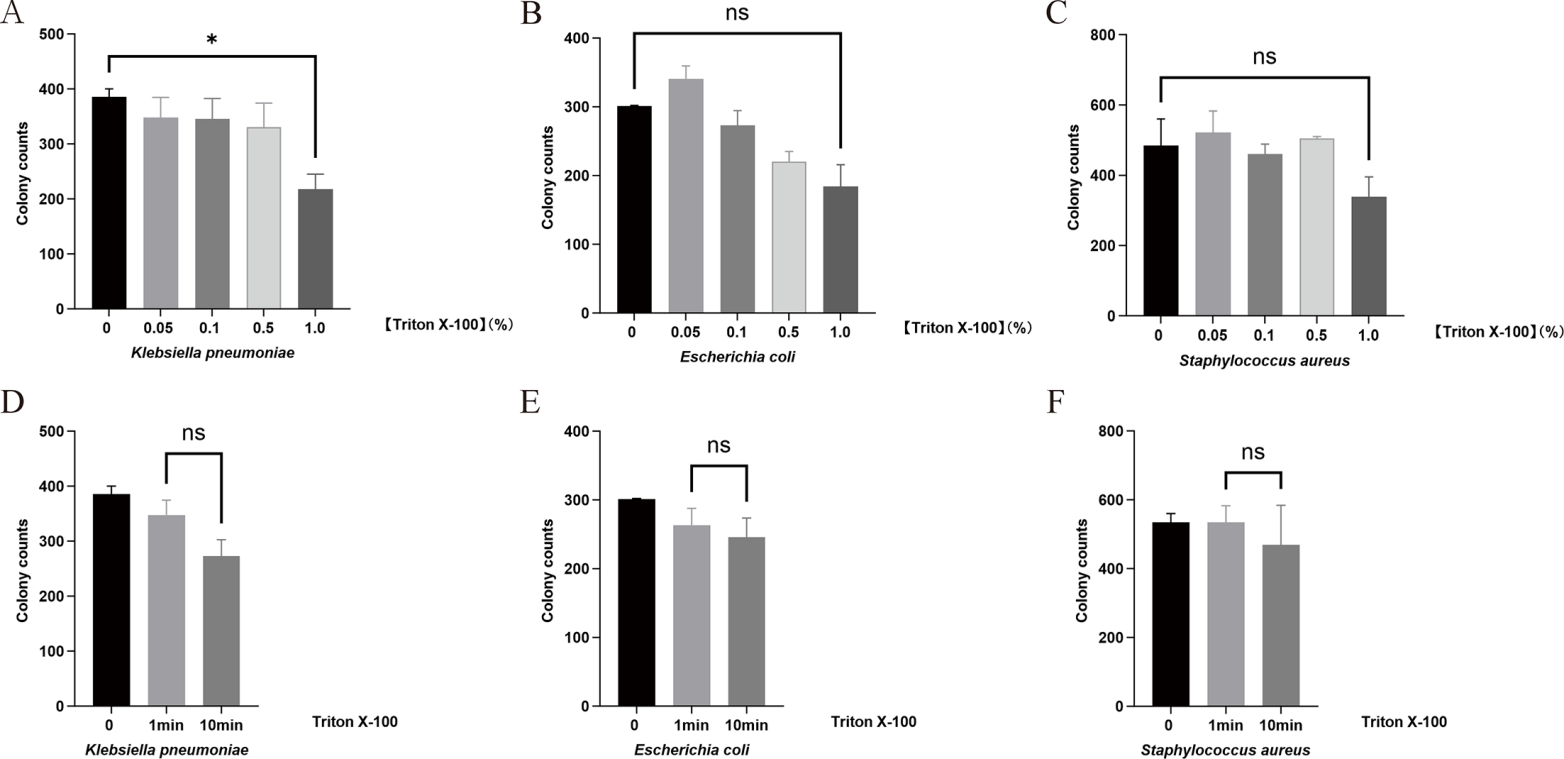
Optimization of Triton X-100 concentration and exposure time for bacterial recovery from ascitic fluid. (**A–C**) Effect of Triton X-100 concentration on growth of *Klebsiella pneumoniae*, *Escherichia coli *and *Staphylococcus aureus*. (**D–F**) Bacterial viability after 1- or 10-min exposure to 0.5% Triton X-100. *Statistical significance: *P* = 0.0165; ns, not significant.

We established a concentration gradient to identify the optimal concentration for effective host cell lysis without compromising bacterial integrity, thereby enhancing detection sensitivity. *Staphylococcus aureus*, *Escherichia coli*, and *Klebsiella pneumoniae*, which are common pathogens in SBP, were selected and treated with different concentrations of Triton X-100 (0.05%, 0.1%, 0.5%, and 1.0%). As shown in Fig. 2A through C, treatment with these four concentrations of Triton X-100 did not result in statistically significant differences in the growth of *Staphylococcus aureus* and *Escherichia coli* (*P* > 0.05). However, for *Klebsiella pneumoniae*, treatment with 1.0% Triton X-100 caused a statistically significant reduction in colony counts compared to untreated controls (mean colonies: 385.5 ± 14.5 vs 217.8 ± 47.7, *P* = 0.0165), indicating bactericidal effects at concentrations ≥1.0%. Additionally, we evaluated the impact of Triton X-100 exposure time on viable bacterial counts. No statistically significant differences were observed between the three bacteria tested across 1-min and 10-min exposure durations (Fig. 2D through F).

Furthermore, we assessed different Triton X-100 conditions using 12 culture-positive ascitic fluid samples (Table S1). Figure S1 shows that the highest number of positive results occurred in groups treated for 1 min at 0.05%, 0.1%, and 0.5% concentrations. Although the difference did not reach statistical significance, the 0.05%–1 min group yielded a numerically higher colony count than the 1.0%–1 min group (*P* = 0.07), indicating a trend that concentrations ≥1.0% may reduce the positivity rate. To ensure robust lysis while maintaining a wide safety margin against potential bacterial damage, we selected 0.5% Triton X-100 with a 1-min incubation as the standard protocol.

### Cohort characteristics and comparative performance of the modified culture method

**Fig 3:**
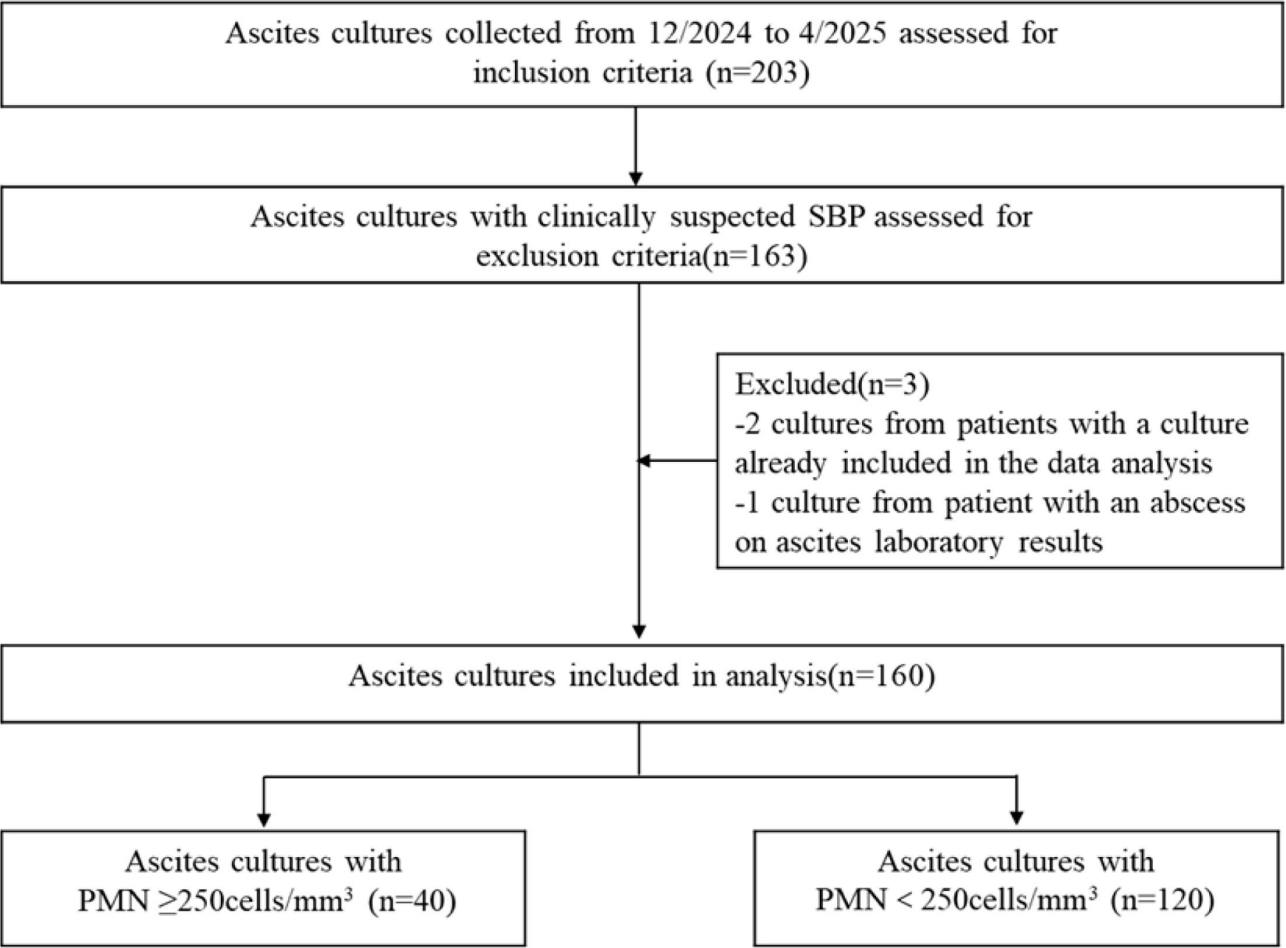
Flowchart of patient selection.

To evaluate the modified bacterial culture method, we analyzed 160 ascitic fluid samples from 160 patients at Beijing Ditan Hospital (Fig. 3). Among the 160 samples, 40 exhibited polymorphonuclear leukocyte (PMN) counts meeting the diagnostic criteria for SBP (≥250 cells/mm³), while the remaining 120 had sub-diagnostic PMN counts (<250 cells/mm³).

Baseline characteristics of the enrolled patients are shown in Table 1. Of the 160 subjects evaluated, the mean age was 60.8 years, and males were predominant (71.9%). The predominant etiology of liver cirrhosis is hepatitis B virus infection (47.5%), followed by alcohol consumption (23.8%). The most common presenting manifestations were fever (46.3%) and abdominal pain (17.5%), with significantly higher prevalence observed in patients with PMN ≥ 250 cells/mm³ compared to those with PMN < 250 cells/mm³ (*P* < 0.05). Abdominal tenderness was present in 41.3% of patients, with hepatic encephalopathy being the most common complication (26.3%), followed by renal insufficiency (14.4%). 93.1% patients received antibiotic therapy within 30 days. The choice of initial antibiotics differed significantly between the two patient groups (*P* < 0.001). Carbapenems were most frequently administered to patients with PMN ≥ 250 cells/mm³ (55%), while β-lactam inhibitor combinations were predominantly used for those with PMN < 250 cells/mm³ (46.8%). Patients with PMN ≥ 250 cells/mm³ had an average hospital stay prolonged by approximately 7 days and a 19.2% increase in mortality rate.

**TABLE 1:** Clinical characteristics of patients^*a*^

Characteristics	Total (*n* = 160)	PMN ≥ 250 cells/mm³ (*n* = 40)	PMN < 250 cells/mm³ (*n* = 120)	*P* value
Age, years	60.8 ± 11.3	57.9 ± 11.8	61.8 ± 11.0	0.057
Male, *n* (%)	115 (71.9%)	36 (90.0%)	79 (65.8%)	0.003
Etiology of cirrhosis, *n* (%)				0.364
Hepatitis B	76 (47.5%)	21 (52.5%)	55 (45.8%)	
Alcoholic	38 (23.8%)	9 (22.5%)	29 (24.2%)	
Autoimmune	16 (10.0%)	3 (7.5%)	13 (10.8%)	
Hepatitis C	6 (3.8%)	0	6 (5.0%)	
MASH	3 (1.9%)	2 (5.0%)	1 (0.8%)	
Others	21 (13.0%)	5 (12.5%)	16 (13.3%)	
Duration of cirrhosis, years	3.9 ± 5.0	4.0 ± 4.4	3.8 ± 5.2	0.810
Time to first decompensation, years	3.7 ± 5.1	3.6 ± 4.4	3.7 ± 5.4	0.924
Clinical manifestations, *n* (%)				
Fever	74 (46.3%)	33 (82.5%)	41 (34.2%)	<0.001
Abdominal pain	28 (17.5%)	12 (30.0%)	16 (13.3%)	0.016
Diarrhea	21 (13.1%)	7 (17.5%)	14 (11.7%)	0.344
Abdominal tenderness	66 (41.3%)	20 (50.0%)	46 (38.3%)	0.194
Hepatic encephalopathy	42 (26.3%)	18 (45.0%)	24 (20.0%)	0.002
Renal impairment	23 (14.4%)	15 (37.5%)	8 (6.7%)	<0.001
GI hemorrhage	17 (10.6%)	7 (17.5%)	10 (8.3%)	0.136
Laboratory findings (blood)				
WBC (×10^9^/L)	6.6 ± 4.5	9.7 ± 6.4	5.6 ± 3.1	<0.001
CRP (mg/L)	34.4 ± 30.3	53.9 ± 39.8	28.4 ± 24.0	<0.001
PCT (ng/mL)	1.3 ± 3.2	3.3 ± 4.8	0.6 ± 2.1	0.002
NLR	9.0 ± 12.0	13.0 ± 11.1	7.6 ± 12.0	0.013
Ascitic fluid				
Total leukocyte count (cells/mm^3^)	1,420.7 ± 4,250.7	4,946.5 ± 7,515.5	245.5 ± 242.6	<0.001
PMN percent (%)	28.2 ± 27.9	69.2 ± 19.9	14.6 ± 12.6	<0.001
PMN count (cells/mm³)	1,022.8 ± 3,698.1	3,995.4 ± 6,608.0	31.9 ± 41.0	<0.001
MELD score	14.9 ± 8.1	20.8 ± 7.6	13.0 ± 7.4	<0.001
Previous use of antibiotics within 30 d	149 (93.1%)	40 (100%)	109 (90.8%)	0.067
Initial antibiotics therapy, *n* (%)				<0.001
BLIC	63 (42.3%)	12 (30.0%)	51 (46.8%)	
Carbapenems	38 (25.5%)	22 (55.0%)	16 (14.7%)	
3rd cephalosporin	32 (21.5%)	3 (7.5%)	29 (26.6%)	
Quinolones	13 (8.7%)	3 (7.5%)	10 (9.2%)	
Others	3 (2.0%)	0	3 (2.7%)	
Combined vancomycin or linezolid	24 (15.0%)	13 (32.5%)	11 (9.2%)	<0.001
Rifaximin administration	40 (25.0%)	10 (25.0%)	30 (25.6%)	0.936
Paracentesis location, *n* (%)				0.003
Inpatient unit	142 (88.8%)	30 (75.0%)	112 (93.3%)	
ICU	18 (11.2%)	10 (25.0%)	8 (6.7%)	
Hospitalization, days	14.7 ± 10.1	19.9 ± 13.4	12.9 ± 8.0	0.003
In-hospital mortality, *n* (%)	17 (10.6%)	10 (25.0%)	7 (5.8%)	0.002

^*a*^ Data were expressed as number (percentage) or mean ± standard deviation. BLIC, β-lactamase inhibitor combination; CRP, C-reactive protein; GI, gastrointestinal; ICU, intensive care unit; MASH, metabolic dysfunction-associated steatohepatitis; MELD, model for end stage liver disease; NLR, neutrophil-to-lymphocyte ratio; PCT, procalcitonin; PMN, polymorphonuclear leukocyte; WBC, white blood cells.

All samples were processed by both the conventional and modified culture methods (Fig. S2). Of the 160 ascitic fluid samples analyzed, conventional culture methods yielded 22 positives, whereas the modified culture method detected 49 positives. This represents a 121.7% increase in the detection rate with the modified method (Table 2). The enhanced performance of the modified culture method was consistently observed in both patient subgroups with PMN counts ≥250 cells/mm³ and those with PMN < 250 cells/mm³, when compared to conventional culture. Furthermore, the modified culture method substantially shortened the time to culture positivity. The mean time to detection was 24 h for the modified method, compared to 2.3 days (55.2 h) for conventional blood culture bottles, constituting a 56.5% reduction in the time required to confirm bacterial growth. Detailed time-to-positivity (TTP) results can be found in Table S2. Critically, the formation of visible colonies on solid media via the modified protocol allows immediate initiation of subsequent bacterial identification and antimicrobial susceptibility testing (AST). In contrast, conventional culture requires subculturing positive broth onto solid agar to obtain isolated colonies before such analyses can begin, adding approximately 24 h of processing time after initial positivity.

**TABLE 2:** Comparative pathogen detection rates and time interval (*n* = 160)

Culture method	Positive/total	Detection rate, % (95% CI)	Mean time (days)
Conventional	22/160	13.8 (8.9–19.6)	2.3
Modified	49/160	30.6 (23.2–37.4)^*a*^	1.0^*b*^

^*a*^ McNemar's *χ*²(1) = 14.26, *P* < 0.001.

^*b*^ *P* < 0.001 by paired *t*-test.

The modified culture method quantified bacterial concentration by enumerating colonies on agar plates after 24 h of incubation. The bacterial concentrations in infected ascitic fluid ranged from 700 colony-forming units (CFU)/10 mL to 1 CFU/10 mL, with a median of 24 CFU/10 mL. The colony counts are presented in Fig. S3. Among the 21 culture-positive ascitic fluid samples with PMN ≥ 250 cells/mm³, 4 exhibited a bacterial concentration of 1 CFU/10 mL, while 7 showed concentrations exceeding 100 CFU/10 mL. Of the 28 culture-positive samples with PMN < 250 cells/mm³, 5 had a bacterial concentration of 1 CFU/10 mL, and 3 demonstrated concentrations >100 CFU/10 mL. Among patients with PMN ≥ 250 cells/mm³, the proportion of samples with bacterial counts >100 CFU/10 mL was higher than that in patients with PMN < 250 cells/mm³ (33.3% vs 10.7%), though this difference did not reach statistical significance (*P* = 0.067).

No statistically significant difference in contamination rate was observed between the methods. Specifically, 10 contaminants (8 *Staphylococcus epidermidis* and 2 *Bacillus* spp.) were identified by conventional culture, compared to 12 (7 *Staphylococcus epidermidis*, 4 *Bacillus* spp., and 1 *Arthrobacter* spp.) by the modified method.

### Microbiological characteristics revealed by conventional and modified culture methods

Optimal antibiotic choice for ascitic fluid bacterial infections is contingent upon accurate identification of the causative species and assessment of its resistance levels; therefore, we next analyzed the distribution and antimicrobial resistance of the cultured bacteria.

All 22 ascitic fluid samples that tested positive using the conventional culture method showed growth of a single microorganism. On the other hand, among the 49 ascitic fluid samples that tested positive using the modified culture method, 39 grew single microorganisms while 10 (20.4%) demonstrated polymicrobial growth (2–3 organisms per sample), including 5 samples with gram-negative/gram-positive combinations. This yielded a total of 62 bacterial isolates from the modified culture method.

Among the bacteria identified by the conventional culture method, *Escherichia coli* was the most prevalent (5 from 22 isolates, 22.7%), followed by *Enterococcus faecium* (4 isolates, 18.2%). In line with the results, of the 62 positive bacteria isolates using the modified culture method, *E. coli* and *Enterococcus faecalis* were the most prevalent (7 isolates each, 11.3%), followed by *E. faecium* (6 isolates, 9.7%) and *Acinetobacter baumannii* (5 isolates, 8.1%). For other clinically relevant isolates, including *Pseudomonas aeruginosa* and *Corynebacterium striatum*, culture results obtained with the modified method were comparable to those generated by the conventional method (Table 3). The conventional culture method detected one anaerobic bacterium (*Clostridium tertium*), which was not identified by the modified culture method.

**TABLE 3:** Microorganisms isolated from ascites by the conventional culture method and the modified bacterial culture method

Organism	No. of episodes (%) that were cultured by			*P* value
	Conventional	Modified	Both	
Gram-negative bacilli	9 (40.9)	30 (48.4)	6 (54.5)	0.49
*Escherichia coli*	5	7	4	
*Acinetobacter baumannii*		5		
*Klebsiella pneumoniae*		3		
*Pseudomonas aeruginosa*	1	1	1	
*Enterobacter cloacae*	1	1	1	
*Klebsiella aerogenes*		2		
*Enterobacter hormaechei*		1		
*Proteus mirabilis*		1		
*Citrobacter freundii*		3		
Acinetobacter sp*.*		2		
Pseudomonas sp*.*	1	3		
*Stenotrophomonas maltophilia*	1	1		
Gram-positive cocci	10 (45.5)	25 (40.3)	5 (45.5)	0.64
*Enterococcus faecalis*	1	7		
*Enterococcus faecium*	4	6	2	
*Staphylococcus haemolyticus*	1	3		
*Staphylococcus hominis*	1			
*Staphylococcus aureus*		1		
*Staphylococcus capitis*		4		
*Staphylococcus epidermidis*		1		
*Streptococcus salivarius*	2	1	1	
*Micrococcus luteus*	1	1		
*Kocuria rosea*		1		
*Corynebacterium striatum*	2	2	2	
*Corynebacterium* spp*.*		2		
*Clostridium tertium*	1			
*Actinomycetes*		1		
*Rothia*		1		
*Brevibacterium*		1		

Next, we conducted antimicrobial susceptibility testing of the cultured bacteria, and the results are summarized in Table 4. Critically, the enhanced sensitivity proved most significant for high-threat pathogens frequently missed by conventional culture methods, especially the WHO-recommended priority antimicrobial-resistant pathogens (24): carbapenem-resistant Enterobacterales (CRE, from 0% to 88.9%), carbapenem-resistant *A. baumannii* (CRAB, from 0% to 100%), and vancomycin-resistant enterococci (VRE; vancomycin-resistant *Enterococcus faecium*, from 25% to 33%; vancomycin-resistant *Enterococcus faecalis*, from 0% to 28.6%). By exposing concealed resistance reservoirs and polymicrobial infections, our method might close critical diagnostic gaps in SBP management, directly enabling survival-improving therapeutic interventions.

**TABLE 4:** Detection rates of drug-resistant bacteria by the two culture methods

Microorganism	Conventional culture method	Modified culture method
Total	4 (14)	33 (46)^*a*^
CRE	0 (6)	16 (18)
CRAB	0 (0)	5 (5)
CRPA	1 (1)	1 (1)
MRSA	0 (0)	0 (1)
MRCNS	2 (2)	7 (8)
VREfs	0 (1)	2 (7）
VREfm	1 (4)	2 (6）

^*a*^ *P* = 0.005 compared with conventional culture method. The numbers in parentheses indicate the total isolates of each bacterial species detected by the conventional/modified culture methods; numbers preceding the parentheses indicate the count of drug-resistant strains among these isolates. CRAB, carbapenem-resistant *Acinetobacter baumannii*; CRE, carbapenem-resistant Enterobacterales spp.; CRPA, carbapenem-resistant *Pseudomonas aeruginosa; *MRCNS, methicillin-resistant coagulase-negative staphylococci; MRSA, methicillin-resistant *Staphylococcus aureus*; VREfm, vancomycin-resistant *Enterococcus faecium*; VREfs, vancomycin-resistant *Enterococcus faecalis*.

### Diagnostic agreement and potential clinical impact of the modified culture method

Given the low baseline positivity rate of conventional ascitic fluid cultures in this cohort (13.8%), the overall positive percent agreement (PPA) for the modified method was inherently limited at 45.5% (Table 5). To further elucidate the diagnostic performance of the modified culture method, we stratified our analysis based on ascitic fluid PMN count, the key criterion for SBP diagnosis.

**TABLE 5:** Overall positive and negative agreement rates between two culture methods in all the patients^*a*^

Modified culture method	Conventional culture method		Total
	Positive	Negative	
Positive	10	39	49
Negative	12	99	111
Total	22	138	160

^*a*^ PPA = 45.5% (95% CI: 24.4%–67.8%), NPA = 71.7% (95% CI: 63.6%–78.8%). NPA, negative percent agreement; PPA, positive percent agreement.

Among samples meeting SBP diagnostic criteria (PMN ≥ 250 cells/mm^3^; *n* = 40), 10 of the 12 ascitic fluid samples positive by conventional culture showed concordant results with the modified culture method. These 10 cases positive by both methods showed largely similar antimicrobial resistance profiles. Among the 12 patients positive by conventional culture, 4 required antibiotic escalation due to unresolved clinical symptoms or persistently elevated inflammatory markers, while 2 de-escalated antibiotics based on susceptibility results. Among the 21 patients who tested positive with the modified culture method, 8 received escalated antibiotic therapy due to unresolved clinical symptoms or persistently elevated inflammatory markers, while 3 had their treatment de-escalated as a result of clinical improvement. We observed that among the 11 patients with positive modified culture but negative conventional culture results, 4 (36.4%) required antibiotic regimen adjustments due to initial treatment failure.

In patients not meeting SBP diagnostic criteria (PMN < 250 cells/mm³; *n* = 120), among the 10 samples positive by the conventional culture method, only one result matched the modified method with the same susceptibility profile (carbapenem-resistant *P. aeruginosa*), while all other findings differed. The conventional method identified three Enterococcus cases, with single instances of *Stenotrophomonas maltophilia*, *Staphylococcus haemolyticus*, *E. coli*, *Micrococcus luteus*, Pseudomonas spp., and Streptococcus spp. Antibiotic escalation was required in four of these cases due to unresolved symptoms. No patient progressed to SBP. Among the 28 patients with ascitic infections confirmed by the modified culture method (yielding 33 isolates), gram-negative bacilli predominated (45.5%, 15/33), including *A. baumannii* (*n* = 4), *E. coli* (*n* = 3), and *Klebsiella pneumoniae* (*n* = 1), followed by gram-positive cocci (39.4%, 13/33) comprising enterococci (*n* = 6) and *S. haemolyticus* (*n* = 3). Clinical resolution (afebrile, alleviation of abdominal symptoms, normalized abdominal examination, or declining inflammatory markers) was achieved in 60.7% (17/28) of cases, while 39.3% (11/28) required antibiotic escalation due to persistent symptoms or elevated inflammatory markers. Notably, 3.6% (1/28) of patients progressed to SBP (PMN ≥ 250 cells/mm³), indicating a potential intervention window at subclinical PMN levels (<250 cells/mm³). Among the 28 patients with positive modified cultures, bacterial load showed no consistent correlation with clinical outcomes. Notably, three patients had high bacterial loads (>100 CFU/10 mL): one with *A. baumannii* required antibiotic escalation due to unresolved symptoms, whereas two (with *E. faecalis* and *Micrococcus luteus*, respectively) achieved clinical resolution with initial therapy. Conversely, among five patients with the lowest bacterial load (1 CFU/10 mL), four required antibiotic escalation due to persistent symptoms.

## DISCUSSION

In the present study, we developed a modified culture method for ascitic fluid involving centrifugation, washing, and Triton X-100-mediated cell lysis. Our results show that this modified method achieved a significantly higher culture positivity rate than conventional bedside inoculation into blood culture bottles for detecting bacterial peritonitis (30.6% vs 13.8%; *P* < 0.0001). The modified culture method also detected bacterial growth more rapidly (1.0 day vs 2.3 days; *P <* 0.001). Bacterial concentrations in infected ascitic fluid ranged from 1 to 700 CFU/10 mL, with a median of 24 CFU/10 mL. Importantly, contaminant detection rates did not differ significantly between the two techniques.

The superior detection capability of our modified method can be attributed to three integral components of the protocol. First, sample concentration overcomes the characteristically low bacterial burden in ascitic fluid—as evidenced by bacterial loads as low as 1 CFU/10 mL in 18.4% (9/49) of culture-positive patients—by enriching bacteria and facilitating the growth of fastidious or slow-growing organisms. Second, the PBS washing step effectively removes residual antimicrobial agents, a critical advantage given that 93.1% of the cohort had recent antibiotic exposure. Although blood culture bottles contain resin and sodium polyanetholsulfonate to absorb or neutralize antibiotics, the inoculation of a large specimen volume results in a lower effective dilution of any residual drugs. Third, Triton X-100 treatment ensures thorough lysis of host cells and cellular aggregates, thereby releasing intracellular bacteria for cultivation.

While saponin is commonly employed as a mild leukocyte-lysing agent in anaerobic blood culture bottles, we selected Triton X-100 for our ascites model primarily due to its more robust and comprehensive lysing capability. This ensures the complete physical disruption of host cells and the release of phagocytosed bacteria, which is critical for viscous, aggregate-prone ascites samples or for cells harboring a high bacterial load (Fig. 1B), conditions under which the pore-forming mechanism of saponin may be insufficient. Furthermore, the efficacy of Triton X-100 is independent of cell type or physiological state, yielding more stable and reproducible results compared to saponin, whose efficiency varies with membrane cholesterol content(25, 26). Moreover, a key distinction of our protocol lies in the sample processing prior to lysis. Conventional inoculation relies on saponin activity within the native, complex ascitic fluid matrix, where proteins, fibrin, and cellular debris may shield target cells or compromise lytic efficiency. In contrast, our method employs centrifugation and washing to concentrate cellular components into a pellet while simultaneously removing soluble inhibitors. This preparatory step enables Triton X-100 to act directly on concentrated targets under optimized contact conditions. Consequently, our integrated strategy—combining sample concentration, washing, and a highly efficient, directly applied lytic agent—specifically addresses the physico-chemical barriers of ascitic fluid, ultimately accounting for the superior bacterial recovery observed in this study.

Positive cultures from both methods were dominated by *E. coli* and enterococci, followed by coagulase-negative staphylococci, streptococci, and *Corynebacterium striatum*—all pathogens commonly implicated in SBP. In addition, the modified culture method increased detection of *A. baumannii* and other Enterobacterales such as Klebsiella spp., *Citrobacter freundii*, and *Proteus mirabilis*, which may relate to antibiotic exposure. At enrollment, 67.8% of patients were receiving β-lactamase inhibitor combinations or carbapenems, antibiotics that more profoundly affect the growth of gram-negative bacilli; alternatively, phagocytic preferences of immune cells within ascites may play a role—an explanation that requires further experimental validation. With the modified method, polymicrobial infections were detected more frequently: among 49 culture-positive ascitic fluid samples, 10 (20.4%) demonstrated polymicrobial growth (2–3 organisms per sample), and 5 (10.2%) involved gram-negative/gram-positive combinations (e.g., *E. coli* plus *E. faecalis*), whereas the conventional method yielded only single-organism growth. This discrepancy is primarily attributable to the methodological differences between the two approaches. In the conventional method, 10 mL of ascitic fluid is inoculated into a single blood culture bottle, where differences in the relative abundance of bacterial species in the specimen, combined with interspecies competition or inhibition during *in vitro* culture, may lead to the detection of only the dominant microorganism. In contrast, our method distributes the processed sample evenly onto solid agar plates, which physically separates different bacteria and facilitates the recovery and identification of multiple species present in the same specimen. Another critical highlight of our study is the enhanced detection of drug-resistant pathogens. Our modified method acts as a spotlight, revealing a hidden reservoir of antimicrobial resistance that conventional cultures consistently miss. The significant increase in the detection rates of CRE, CRAB, and VRE is of paramount clinical importance. Failure to identify these resistant organisms directly leads to inadequate initial empirical therapy, which can ultimately result in treatment failure.

Among patients with PMN ≥ 250 cells/mm^3^, agreement between the two methods was good. Of the 12 ascitic fluid samples positive by the conventional method, 10 completely matched the modified method. In two discordant cases, one yielded *Staphylococcus hominis* by the conventional method but *E. faecalis* by the modified method (bacterial load 3 CFU/10 mL), suggesting that at low inocula, distinguishing pathogens from contaminants can be challenging; in the other, the conventional method recovered the anaerobe *Clostridium tertium*, which the modified method failed to detect. Although the modified workflow also included anaerobic culture, its multiple handling steps may have led to bacterial death. Among patients with PMN < 250 cells/mm^3^, concordance was poor: only a single case of *P. aeruginosa* was detected by both methods, and all other results were discordant. This likely reflects lower bacterial concentrations in ascites in this subgroup (among the 28 culture-positive samples by the modified method, only 3 had >100 CFU/10 mL). In such cases, culture results may be influenced by sampling heterogeneity or uneven intraperitoneal antibiotic distribution. This subgroup represents a true “diagnostic gray zone.” Our modified method identified a substantial number of infections here (28 cases), and subsequent clinical data revealed that a proportion of these patients subsequently required antibiotic escalation or even progressed to overt SBP. This strongly suggests that in patients with clinical suspicion of infection but sub-diagnostic PMN counts, the modified culture method holds potential for early warning and intervention. These findings may call for a re-evaluation of the diagnostic and clinical significance of “bacterascites.”

The strength of our study lies in addressing a growing clinical dilemma: the declining yield of conventional blood culture bottles in an era of escalating empirical antibiotic use (27). We are the first to specifically investigate and provide an effective solution for this challenging patient population. Moreover, this study provides the first direct microscopic visualization of bacterial phagocytosis by immune cells in ascitic fluid from patients with SBP. Following membrane lysis treatment, these phagocytosed bacteria demonstrated cultivability, confirming their metabolic activity and proliferative capacity. This finding not only offers direct evidence that “phagocytosis-mediated bacterial sequestration” constitutes a key mechanism underlying culture-negative samples but also establishes an experimental foundation for further investigation into SBP pathogenesis and bacterial immune evasion strategies. However, our findings must be considered alongside several limitations. First, although the modified culture method demonstrated superior sensitivity for most pathogens, it failed to recover *Clostridium tertium*—an anaerobic bacterium detected by the conventional method. This was likely due to the susceptibility of fastidious anaerobes to the multiple processing steps involved. Second, this was a single-center study, and the sample processing protocol, while effective, is more labor-intensive than standard bedside inoculation and requires specific laboratory expertise. It is noteworthy, however, that despite the increased handling, the contamination rate did not differ significantly from that of conventional cultures, reinforcing the robustness of our protocol. Future multi-center, prospective studies are warranted to validate the cost-effectiveness of this method and, most importantly, to investigate its impact on hard clinical endpoints such as patient mortality and hospital length of stay.

In conclusion, our integrated centrifugation-washing-Triton X-100 lysis protocol successfully addresses a core bottleneck in the microbiological diagnosis of SBP by systematically overcoming the barriers of phagocytic sequestration and antibiotic interference. This approach delivers a dual clinical value: significantly enhancing diagnostic accuracy by uncovering more true positives, polymicrobial infections, and concealed resistant pathogens, and substantially accelerating the diagnostic timeline by enabling same-day colony growth for immediate identification and susceptibility testing. Looking forward, we envision this modified culture method being integrated into future SBP diagnostic algorithms. Its implementation holds great promise for guiding timely, targeted antimicrobial therapy, thereby directly contributing to improved antimicrobial stewardship and patient outcomes in this vulnerable population.

### Conclusion

In conclusion, our novel culture method enhances SBP diagnosis by overcoming phagocytosis and antibiotic interference. It improves pathogen detection, reveals hidden resistances, and speeds up results, promising to optimize targeted therapy and patient management.

## MATERIALS AND METHODS

### Patients and samples enrolled in this study

This study included adult patients with cirrhosis-related ascites who were clinically suspected to have SBP and admitted to the Hepatology Department, Department of Integrated Traditional Chinese and Western Medicine, and Intensive Care Unit of Beijing Ditan Hospital, Capital Medical University, between December 2024 and April 2025. Clinical suspicion of SBP was defined as the presence of either local manifestations of peritonitis, like tenderness in the abdomen, abdominal pain, vomiting, or ileus, or systemic inflammation signs, such as fever or hypothermia, chills, a change in white blood cell count, hepatic encephalopathy, worsening liver function, worsening renal function, and gastrointestinal hemorrhage (2).

Exclusion criteria comprised secondary bacterial peritonitis (confirmed by surgical evidence of gastrointestinal perforation or radiographic abscess), current peritoneal dialysis, non-cirrhotic ascites etiologies (such as cardiogenic ascites, nephrogenic ascites, or pancreatic ascites), and cases with incomplete clinical data (Fig. 3).

For each enrolled patient, the first 60 mL ascitic fluid sample collected after hospital admission was processed using a standardized protocol: 20 mL was equally aliquoted into aerobic and anaerobic blood culture bottles (BD BACTEC Plus Aerobic/F Culture Vials and BD BACTEC Lytic/10 Anaerobic/F Culture Vials) at the bedside, while the remaining 40 mL was transferred into a sterile tube and promptly transported to the clinical microbiology laboratory at 4°C within 2 h for modified culture.

This study was approved by the Ethics Review Committee of Beijing Ditan Hospital, Capital Medical University. Patients gave written informed consent before entry into the study.

### Conventional culture method

The conventional culture method involves bedside inoculation of 10 mL ascitic fluid into each of the paired aerobic and anaerobic blood culture bottles (BD BACTEC Plus Aerobic/F Culture Vials and BD BACTEC Lytic/10 Anaerobic/F Culture Vials). BD BACTEC Lytic/10 Anaerobic/F contains soy and casein digestion broth enriched with CO_2_ and N_2_ atmosphere, as well as saponin and sodium polyanetholsulfonate as novel elements that act as a lytic agent and inhibit bactericidal activities in the blood, especially the growth of anaerobic bacteria. BD BACTEC Plus Aerobic/F is made up of soy and casein digestion broth enriched with a CO_2_ atmosphere and antibiotic neutralizing resins (28).

The samples were incubated in the automated BD BACTEC FX system (Becton Dickinson) at 37°C for 5 days, after which they were considered negative. The instrument performed automated readings every 10 minutes (28). Upon a positive alarm, patient information was verified, and the TTP was recorded. Subsequently, 2–3 drops of the bacterial broth were subcultured onto blood agar plates and immediately placed in appropriate incubation conditions (35°C, aerobic/CO_2_/anaerobic) for 48 h. The plates were examined twice daily for growth. The isolated pathogens were then identified by MALDI-TOF mass spectrometry; antimicrobial susceptibility testing was performed simultaneously by the VITEK 2 system (bioMérieux, Marcy-l'Étoile, France) or, when indicated, by Etest strips (AB Biodisk, Solna, Sweden). All procedures strictly adhered to the guidelines established by the Clinical and Laboratory Standards Institute (CLSI).

### Modified culture method

In the modified culture process, 20 mL of ascitic fluid was centrifuged at 8,000 rpm for 3 min, washed once with PBS, treated with 0.5% Triton X-100 for 1 min, and recentrifuged. The final pellet was resuspended in ~100 µL and inoculated equally onto chocolate agar and blood agar. The plates were incubated under a 5% CO_2_ atmosphere at 35°C and examined twice daily for 48 h.

Meanwhile, an additional 20 mL of ascitic fluid was subjected to the identical procedure, and the final suspensions were plated onto blood and chocolate agar plates. These plates were immediately placed into a 2.5 L anaerobic jar equipped with a 2.5 L anaerobic gas producing bag and an oxygen indicator; the indicator color change confirmed the absence of oxygen. Plate inoculation and jar sealing were completed within 1 min. The jars were then incubated at 35°C and inspected twice daily for bacterial growth.

Observed colonies were immediately subjected to 16S rRNA gene (primers: 27F: AGAGTTTGATCCTGGCTCAG; 1492R: GGTTACCTTGTTACGACTT), while plates showing no bacterial growth after 48 h were discarded. AST was primarily performed using the disk diffusion method on Mueller-Hinton agar in accordance with CLSI M02-Ed13 guidelines (Clinical and Laboratory Standards Institute, 2023). When isolates exhibited ambiguous inhibition zones, when disk diffusion was technically unreliable, or when precise minimum inhibitory concentration (MIC) values were required for clinical decision-making, MIC determination was carried out by broth microdilution following CLSI M07-Ed12 (Clinical and Laboratory Standards Institute, 2023). All AST results were interpreted using CLSI M100-Ed34 clinical breakpoints (Clinical and Laboratory Standards Institute, 2024).

### Establishment and optimization of the modified culture method

To determine the optimal concentration of Triton X-100 and the appropriate processing time, single colonies of *Escherichia coli*, *Staphylococcus aureus*, and *Klebsiella pneumoniae* isolated from ascitic fluid were selected and inoculated into 5 mL Luria-Bertani (LB) liquid medium. After incubation at 37°C with shaking at 200 rpm for 6–8 h, the bacterial suspensions were adjusted to 5 × 10⁴ CFU/mL using a 96-well plate reader (BioTek Epoch 2, USA). Specifically, OD_600_ values were measured at 600 nm, and the target density (5 × 10⁴ CFU/mL) was achieved by a two-step 10-fold dilution (10^−2^ and 10^−4^) based on species-specific calibration curves established previously (1 OD_600_ ≈ 8 × 10⁸ CFU/mL for *E. coli*, 6 × 10⁸ CFU/mL for *K. pneumoniae*, and 3 × 10⁸ CFU/mL for *S. aureus*). Subsequently, 10 μL aliquots of each suspension were mixed with 1 mL of Triton X-100 solutions at various concentrations (0.05%, 0.1%, 0.5%, and 1.0%) and treated for different durations (1 min and 10 min). Following centrifugation, the final pellets were resuspended in approximately 100 μL and plated onto LB agar plates. The plates were incubated at 37℃ overnight, and colony-forming units (CFU) were enumerated.

Additionally, we selected patients with a large volume of ascitic fluid and positive cultures, collected an additional 80 mL of ascitic fluid, and processed it according to the modified culture method. After centrifugation and washing with PBS, the samples were treated with different concentrations of Triton X-100 (0.05%, 0.1%, 0.5%, and 1.0%) for varying durations (1 min and 10 min), followed by plating onto chocolate agar plates. The plates were incubated overnight at 37°C, and colony-forming units were enumerated.

All Triton X-100 solutions were prepared with PBS as the solvent.

Because the bacterial concentration in ascitic fluid is extremely low, the number of colonies grown on culture plates is not used as a criterion for determining contamination. An organism is regarded as a contaminant if it represents a common laboratory contaminant and the patient shows clinical improvement in the absence of antibiotic therapy. Common contaminating organisms in our laboratory include Bacillus spp., Cutibacterium spp., Lactobacillus spp., and *Staphylococcus epidermidis*.

### Statistical analysis

Continuous variables were presented as mean ± standard deviation if normally distributed, or as median with interquartile range if not. Categorical variables were expressed as numbers and percentages (%). The normality of data distribution was assessed using the Shapiro-Wilk test.

For the comparison of culture positivity rates and the detection of polymicrobial infections between the conventional and modified culture methods, McNemar’s test and the chi-square (*χ*²) test were employed as appropriate. The Mann-Whitney *U* test (for non-normally distributed data) or the independent samples *t*-test (for normally distributed data) was used to compare continuous variables, such as time-to-detection and colony-forming units (CFU), between the two methods and across patient subgroups. The agreement between the two culture methods was evaluated by calculating the PPA and negative percent agreement (NPA).

All statistical analyses were performed using the Statistical Package for the Social Sciences (SPSS, version 27.0) or GraphPad Prism (version 9.0). A two-tailed *P* <0.05 was considered statistically significant for all analyses.

## Data Availability

The data that support the findings of this study are available from the corresponding author upon reasonable request.

## SUPPLEMENTAL MATERIAL

Supplemental material spectrum.03435-25-s0001.pdf Fig. S1 to S3; Tables S1 and S2.

ASM does not own the copyrights to Supplemental Material that may be linked to, or accessed through, an article. The authors have granted ASM a non-exclusive, world-wide license to publish the Supplemental Material files. Please contact the corresponding author directly for reuse.
