# Supplementary material for: An integrated centrifugation-washing-lysis method significantly improves microbiological diagnosis of spontaneous bacterial peritonitis
Source: Microbiol Spectr. 2026 Mar 16;14(4):e03435-25. doi: 10.1128/spectrum.03435-25 (PMC13055387; doi:10.1128/spectrum.03435-25)
Supplement: Supplemental material — Fig. S1 to S3; Tables S1 and S2. [file spectrum.03435-25-s0001.pdf]

Patient ID	Triton X -100							
	0.05% 1min	0.1% 1min	0.5% 1min	1.0% 1min	0.05% 10min	0.1% 10min	0.5% 10min	1.0% 10min
DT115	26	3	1	3	0	0	0	0
DT121	1	0	0	0	0	0	1	0
DT122	1	1	4	0	4	4	0	3
DT124	4	2	3	0	5	2	0	2
DT127	25	25	17	9	16	20	10	6
DT132	2	0	3	0	2	1	0	0
DT145	39	29	22	44	77	38	46	26
DT169	1	0	0	0	0	0	0	0
DT181	0	1	1	0	0	4	1	1
DT186	1	0	0	0	0	0	0	0
DT203	0	0	4	1	0	0	1	2
DT205	1	1	1	0	0	0	0	0

Table S1 The number of bacterial colonies grown on chocolate agar plates from ascites samples of 12 culture-positive patients after treatment with Triton X-100 at various concentrations and exposure times.

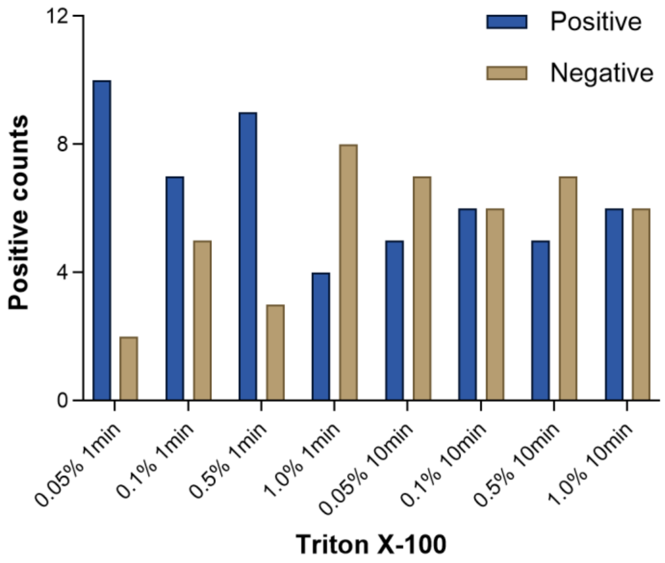

Figure S1. Number of culture-positive results from 12 patients after treatment with Triton X-100 at various concentrations and exposure times.

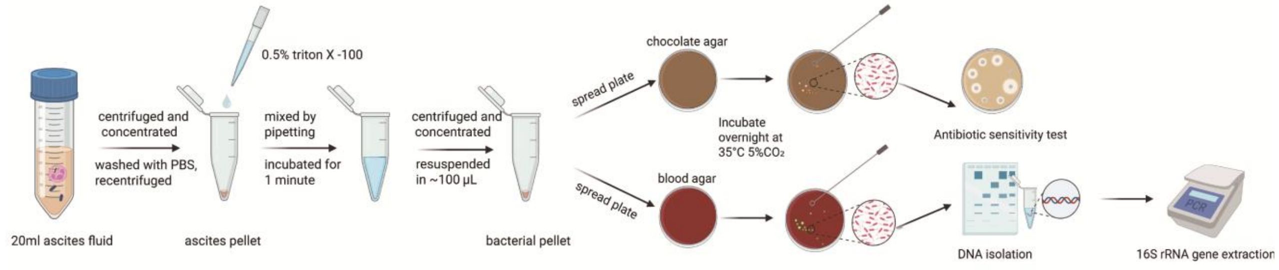

Figure S2. Flowchart of the Modified Culture Protocol

Time	No. of patients with positive culture results by:	
	Modified	Conventional
12h	10	0
12~24h	30	2
24~48h	9	13
48~72h	0	5
> 72h	0	2

Table S2. Time from inoculation to positivity.

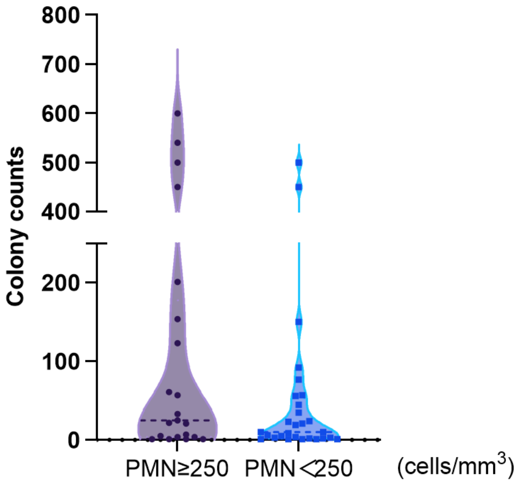

Figure S3. Colony counts (CFU/plate) for individual patients, stratified by PMN level.
